# Insects allocate eggs adaptively according to plant age, stress, disease or damage

**DOI:** 10.1098/rspb.2022.0831

**Published:** 2022-07-13

**Authors:** Lachlan C. Jones

**Affiliations:** School of Biological Sciences, The University of Queensland, Brisbane 4072, Australia

**Keywords:** host plant, oviposition, preference–performance, quality, survival, vigour

## Abstract

Most herbivorous insects can only survive on a small subset of the plant species in its environment. Consequently, adult females have evolved sophisticated sensory recognition systems enabling them to find and lay eggs on plants supporting offspring development. This leads to the preference–performance or ‘mother knows best’ hypothesis that insects should be attracted to host plants that confer higher offspring survival. Previous work shows insects generally select plant species that are best for larval survival, although this is less likely for crops or exotic host plants. Even within a species, however, individual plants can vary greatly in potential suitability depending on age, access to water or nutrients or attack by pathogens or other herbivores. Here, I systematically review 71 studies on 62 insect species testing the preference–performance hypothesis with sets of plants varying in age, stress, fungal/microbial infection or herbivore damage. Altogether, 77% of insects tested with a native host (*N* = 43) allocated their eggs to plants best for offspring development, as did 64% (*N* = 22) of insects tested with an exotic host. Results were similar across plant age, stress, disease and damage categories. These findings show adaptive maternal behaviour in insects occurs for both host species and variation among individual plants.

## 1. Introduction

Most herbivorous insects begin life as an egg, which its mother typically provisions with food for when it hatches by placing it on an edible plant. Insects in the larval stage predominantly feed and grow, and it is only after undergoing metamorphosis that they develop wings [1]. The food requirements and limited dispersal abilities of most insect larvae means their survival depends on the oviposition host of their mothers. This should in theory lead to selection on female insects to lay their eggs on plants conferring high larval survival [2], a prediction commonly called the preference–performance hypothesis [3,4].

Hundreds of papers have been published over the last few decades testing the relationship between relative attractiveness of a set of host plants for egg-laying and larval survival rates on these hosts, and the hypothesis has been the subject of three systematic reviews. Mayhew [3] and Gripenberg *et al*. [4] found that although a positive relationship between female egg-laying and larval survival was the most common test outcome, a significant proportion of studies (45% of the 133 studies reviewed by [3]) found egg-laying was not well correlated with larval performance. In the most recent systematic review, however, I showed that most cases of poor relationships between oviposition and offspring survival across host plants involved tests of cultivated crops or plants originating outside the insect's native range. When considering only the experiments testing native host plants, 83% of insect species allocated eggs to the best or equal-best host plants for their offspring [5]. The predominance of positive oviposition–performance relationships held across all the major insect orders and was just as true for generalist insect species as specialists.

The 178 studies I included in [5] were restricted to tests of egg-laying and larval survival across a set of host species. Yet many studies have investigated whether ovipositing females also discriminate among conspecific host plants in different physical condition, whether as a result of age, stress, herbivore damage, disease or symbiotic association. Such factors can certainly affect larval survival rates, with some insects performing better on stressed plants, perhaps because of increased soluble nitrogen levels or reduced defences (plant stress hypothesis) [6], while others perform better on healthy plants or plant modules that grow rapidly (plant vigour hypothesis) [7]. Whether plant stress has a beneficial or harmful effect on herbivores is highly dependent on the type of stress and the insect's mode of feeding [8]. Infection of plants by fungi (pathogenic or mutualistic) may also have variable effects, although a recent meta-analysis of 101 papers suggests fungal infected plants were in general less attractive to insect herbivores and conferred reduced insect survival rates [9]. Most of the individual studies in this analysis did not address preference–performance correlations, however, testing preference or performance of a particular herbivore but not both.

When it comes to oviposition, the cues an insect might use to recognize healthy or stressed/damaged/diseased plants are likely to differ from those used to recognize host species, and we should not assume the preference–performance hypothesis holds both ways. Indeed, tests with the European butterflies *Polygonia c-album* and *Vanessa cardui* suggest that although they allocate more eggs to plant species suitable for their offspring [10,11], they did not distinguish between good and poor quality plants of the same species [12].

**Figure 1.**
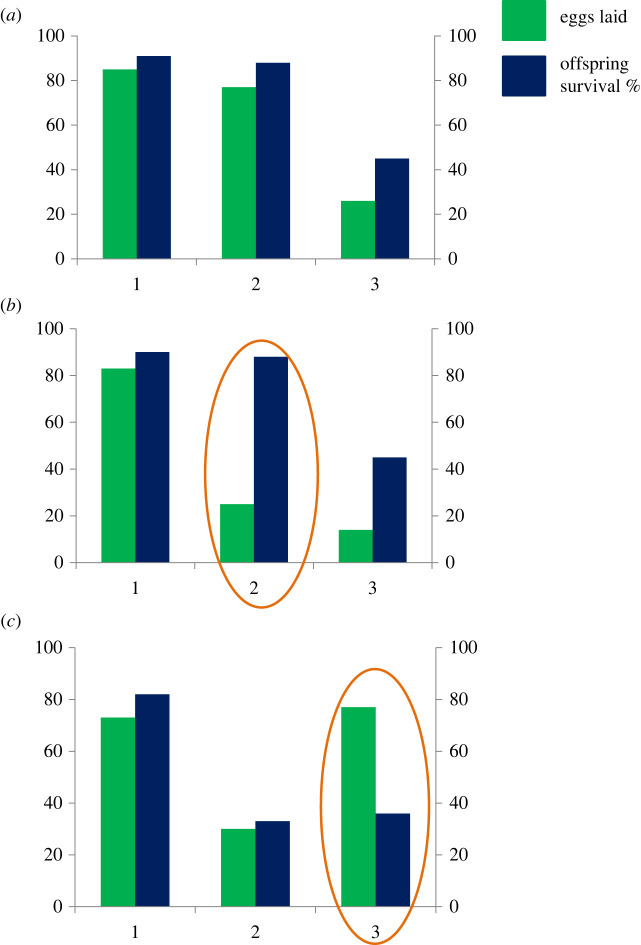
Hypothetical situations involving oviposition and survival of an insect species across three host plant treatments (1–3) that would be described as (*a*) positive relationship: numbers of eggs laid match survival rates, (*b*) few eggs laid on high survival host: plants 1 and 2 are equally suitable but plant 2 (indicated within an ellipse) receives fewer eggs or (*c*) many eggs on low survival host: plant 3 (indicated within an ellipse) is worse than 1 or 2 but receives as many eggs as the best plant. (Online version in colour.)

In this study, I aimed to determine the level of support for the preference–performance hypothesis when comparing insect oviposition and survival across plants with and without damage, water or nutrient stress, disease, or comparing young to old plants. Outcomes of individual studies I classed as *oviposition matches survival* (positive relationship), *few eggs on high survival host* or *many eggs on low survival host* (figure 1). This allowed me to answer three main questions. (i) Do most insects allocate their eggs adaptively when ovipositing on plants with varying age, damage, water or nutrient stress or disease infestation? (ii) Are insects more likely to respond adaptively to the condition of their native hosts compared to exotic or crop host species? (iii) Is a positive preference–performance relationship more common for specialist insects?

## 2. Material and methods

### (a) Literature search methods

I found several studies by looking through the first 1000 results (the maximum viewable) in a basic search in Google Scholar with the keywords: oviposition, preference, performance, survival, host and plant. I completed this on 20 April 2020. I also performed an advanced search in Web of Science: Topic = (preference OR oviposition) AND Topic = (performance OR survival) AND Topic = insect. This yielded 3065 results up until the end of 2020, which I finished searching through on 16 January 2021. I tracked down further studies using the appendices and citing literature of Mayhew [3] (497 citations) and Gripenberg *et al*. [4] (655 citations) as of 11 January 2021. To be included, a study must have counted numbers of eggs laid across the plant treatments in the laboratory or field, and included a test of survival rates in their measures of offspring performance. I did not include studies comparing different parts of the same individual plant except for galling or mining insects, because more mobile larvae can move between leaves or flowers on an individual plant, reducing the selection pressure on oviposition site at this level. I also excluded studies comparing oviposition to survival across a set of individual plants without qualifying what factors (if any) differed across the test plants.

### (b) Extraction of data from studies

For each insect species in studies that met these criteria, I recorded whether a positive relationship was detected between preference and performance and, if not, whether the species fell into the ‘few eggs on high survival host’ or ‘many eggs on low survival host’ categories (figure 1). To be labelled as showing a positive relationship, offspring survival in the study (or other proxy measures of fitness such as pupal weight or shorter development times, if survival did not differ significantly) must have been higher on host plants receiving significantly more eggs than another, or similar on hosts receiving equal numbers of eggs. If, however, two or more hosts conferred the same offspring fitness but one received significantly more eggs than another, then the study was labelled ‘fewer eggs on high survival host’. And if a plant significantly worse for offspring fitness (in terms of either survival rates or pupal mass/adult fecundity) received a similar number or more eggs than a more suitable host plant, the study was labelled ‘many eggs on low survival host’ (figure 1). I did not consider slower development time alone as sufficient grounds for applying the ‘many eggs on low survival host’ label if survival rates and pupal weights were not shown to differ, because there is little evidence for the hypothesis that a prolonged larval stage equals higher mortality from natural enemies in the field [13,14]. However, a significantly slower development time was considered in cases where mean survival on the slower growth host was lower but not quite at statistical significance.

I grouped studies on a particular insect species into four categories depending on the type of variation in plant condition that it was being tested against. These categories were age/phenology, stress (water, nutrients, shading etc.), disease or symbiont infestation and herbivore damage. I also divided the studies depending on whether the test host plant was native to the insect's native range, as I did in [5]. Domesticated crops, fruit trees and ornamentals I considered non-native regardless of the origins of their wild ancestors. I also divided insect species by specialization, with species specialists, genus specialists, family specialists, insects using 2–3 families and generalists (four or more families or two or more plant phyla).

### (c) Statistical comparisons

I regarded each insect species as a replicate within a test category. I compared proportions of species with the outcomes ‘Oviposition matches survival’, ‘Few eggs on high survival host’ and ‘Many eggs on low survival host’ across the four test categories (age/size/phenology, stress, disease and damage) and comparing native and non-native plants using chi-square or Fisher's exact tests where appropriate (table 1). I also compared results for insect species from monophagous to generalist (table 2). I favoured a vote counting method over meta-analysis because the data found in most preference–performance studies do not allow calculation of effect sizes for a meta-analysis, a problem raised by [4]. For insect species tested more than once within the same test category, I used the most common experimental outcome if there was one. If there had been two tests with different outcomes, I conservatively assigned the species the negative result (e.g. a species that showed ‘Few eggs on high survival host’ in one test and ‘Many eggs on low survival host’ in another was taken as ‘Many eggs on low survival host’). When presenting data pooled across test categories, those individual species with conflicting test results in different categories (e.g. positive and many eggs on low survival host) were divided evenly between the counts for each test outcome. If there was no other species with this combination of outcomes across categories, the more common outcome (if any) was used, otherwise the more negative outcome. The actual results with species tested more than once can be seen in the outcome consistency section (table 3).

**Table 1.** Number of insect species (with percentages of total) showing positive (oviposition matches survival), few eggs on high survival host and many eggs on low survival host test outcomes for native and non-native test plants in each category of intraspecific plant variation. Combined results are given in the bottom table, with each species only counted once (see Methods: statistical comparisons). The *p*-values of Fisher's exact tests are given to the right of each contingency table, none of which showed a significant difference across native and non-native test plants.

native range	positive	few eggs high survival host	many eggs low survival host	*p*-value
plant age, size or flowering state				
yes	9 (39%)	6^a^ (26%)	8^a^ (35%)	*p* = 0.37
no	5 (62%)	0	3^b^ (38%)	
plant stress				
yes	10 (59%)	3 (18%)	4 (24%)	*p* = 0.33
no	3 (30%)	2^c^ (20%)	5 (50%)	
plant disease				
yes	2 (50%)	2 (50%)	0	*p* = 0.37
no	3 (60%)	0	2 (40%)	
herbivore damaged plants				
yes	3 (43%)	3 (43%)	1 (14%)	*p* = 0.76
no	5 (71%)	1 (14%)	1 (14%)	
overall				
yes	21 (49%)	12 (28%)	10 (23%)	*p* = 0.20
no	12 (55%)	2 (9%)	8 (36%)	

^a^One of each of these possibly explained by enemy-free space.

^b^One case possibly due to adult-deterring trichomes on older host plants (which were better for offspring).

^c^One of these possibly explained by the lack of chemical defence.

**Table 2.** Numbers of insect species at each level of host plant specialization for which female oviposition matched larval survival on particular plants, laid few eggs on high survival host or many eggs on low survival host plant (table 3 for how species with inconsistent results were assigned).

extent of specialization test outcome	species specialist	genus specialist	family specialist	2–3 families	generalist
native host plants					
oviposition matches survival	4	5	9	1	2
few eggs high survival host	3	3	6	0	0
many eggs low survival host	3	4	1	1	1
non-native host plants					
oviposition matches survival	n/a	2	5	2	3
few eggs high survival host	n/a	0	1	0	2
many eggs low survival host	n/a	0	1	2	4

**Table 3.** Results for those insect species tested more than once in either the same or different test category. ✓ = positive relationship (oviposition matches survival), ● = few eggs on high survival host and ✗ = many eggs on low survival host. The symbol in brackets immediately right of the species name indicates how each species was counted towards the overall totals in table 1. Note that for purposes of tallying, for example, in the case of *Panolis flammea* (✓) and *Bemisia tabaci* *Q* (✗), both of which had one positive and one ‘many eggs on low survival host’ result, the assigning of ✓ to *P. flammea* and ✗ to *B. tabaci* rather than vice versa was arbitrary.

specialization	species (overall classing)	age	stress	damage	disease
native hosts					
genus	*Neodiprion sertifer* (✗)	✗	✓		
genus	*Eurosta solidaginis* (●)		●	●●	
family	*Pieris brassicae* (●)	●●			
family	*Chromatomiya milii (*✓)	✗	✓✓		
family	*Euchloe hyantis* (●)	●		✓	
family	*Coenonympha hero* (●)				●●
generalist	*Vanessa cardui (*✓)	✗	✓		
generalist	*Polygonia c-album* (✗)	✓✗	✗		
generalist	*Samea multiplicalis (*✓)		✓	✓●	
non-native hosts					
generalist	*Apolygus lucorum* (✓)	✓✓✓			
generalist	*Liriomyza trifolii* (●)	✗	✓●		
generalist	*Otiorhynchus sulcatus* (✗)	✗		✗	
generalist	*Panolis flammea* (✓)	✗		✓	
generalist	*Bemisia tabaci B* (✓)	✓			✓✓✓✓✓✗
generalist	*Spodoptera exigua* (✗)		✗✗●		✓
generalist	*Epiphyas postvittana* (✗)				✗✗
generalist	*Bemisia tabaci Q* (✗)				✗✓
family	*Chilo partellus* (✗)		✓✓✗✗✗		
family	*Plutella xylostella* (✓)		✓✓		
family	*Eldana saccharina* (✓)		✗		✓

To control for possible confounding effects of phylogeny, I paired each of the 18 generalist insect species (more than one host family) with the closest possible related specialist within its order (with the condition that a specialist species could not be paired more than once). If more than one equally closely related candidate pair was available, I selected one using random.org [15]. I likewise created 19 pairs of species related at least to the level of order in which one was tested with a native host plant, one an exotic host. Phylogenetic pairing was achieved with reference to [16–21]. For each pair, I determined the ‘winner’ based upon test outcome(s) for each species, with positive relationship > few eggs on high survival host > many eggs on low survival host. I used the most common outcome for species tested several times. If tested twice with different outcomes, I considered it to sit between the two, such that *Positive/Many eggs low survival host* would win over a species with a single test showing *Many eggs on low survival host* but lose to a species with a single *Positive* relationship. Proportions of pair winners belonging to native/exotic and generalist/specialist were compared with binomial tests, with ties allocated evenly.

## 3. Results

Altogether my search yielded 71 relevant studies involving 62 insect (29 Lepidoptera, 10 Coleoptera, nine Hymenoptera, eight Hemiptera and six Diptera) and 65 plant species (10 Poaceae, seven Brassicaceae, seven Salicaceae, five Solanaceae, three Asteraceae, three Cucurbitaceae, two Cupressaceae, two Ericaceae, two Fabaceae, two Malvaceae, two Pinaceae, two Rosaceae, one Amaranthacae, one Amaryllidaceae, one Anacardiaceae, one Annonaceae, one Apiaceae, one Balsaminaceae, one Euphorbiaceae, one Lamiaceae, one Lygodiaceae, one Myrtaceae, one Passifloraceae, one Plantaginaceae, one Polygonaceae, one Salviniaceae, one Ulmaceae, one Urticaceae, one Verbenaceae and one Vitaceae).

### (a) Native and non-native hosts: are most insects allocating eggs adaptively?

Overall, most insects tested with plants of varying conditions from their native range (*N* = 43) allocated most of their eggs to either the host best for their offspring (Oviposition matches survival, 49%) or to a subset of two or more hosts conferring equal highest offspring survival (Few eggs on high survival host, 28%). This leaves just under a quarter (23%) laying a similar or greater number of eggs on a worse host plant for offspring survival (Many eggs on low survival host) (table 1).

For insects tested with non-native host plants (*N* = 22), 12 species (55%) had oviposition matching survival, two (9%) laid few eggs on high survival host(s) and eight (36%) laid many eggs on low survival host(s). These proportions were not significantly different from those seen in tests with native hosts (Fisher's exact test: *p* = 0.20). Controlling for phylogeny through pairing related species (sometimes the same species) tested with native and exotic host plants (see electronic supplementary material) likewise did not find significantly more adaptive oviposition in tests with native hosts, with nine native winners, four ties and six exotic winners (binomial test, *p* = 0.65).

### (b) Are there differences in responses to stress, disease, damage and age variable plants?

The proportions of insect species with apparently maladaptive oviposition behaviour (many eggs on low survival host) on their native host plants ranged from zero when tested with diseased plants to 35% when tested with hosts varying in age, size or flowering state. The proportion of species tested with native hosts showing each test outcome did not differ significantly across the test categories (Fisher's exact test, *p* = 0.55). Plant age/phenology (*N* = 23) and plant stress (*N* = 17) had been tested more extensively than disease (*N* = 4) or damage (*N* = 7), however. Pooling tests with native and non-native host plants together increased sample sizes a little (table 1), but there were still no differences across test categories in the proportions of insects showing positive, ‘few eggs on high survival host’ or ‘many eggs on low survival host’ test outcomes (Fisher's exact test, *p* = 0.51).

### (c) Are more specialized insects better at selecting plants with high larval survival?

The 43 insect species tested with plants from their native range can be divided into 10 species specialized on one plant species, 12 specialized on one genus, 16 on one family and five with host plants across more than one family, including three with hosts across four or more families or at least two phyla (generalists) (table 2). Family specialists most closely followed predictions of the preference–performance hypothesis, with all but one showing either a positive or few eggs on high survival host outcome (table 2). However, the differences in test outcome across host range were not significant (Fisher's exact test: *p* = 0.56). Species specialists could not, by definition, be tested with any plant but their native host; however, there were tests with non-native host plants with two genus specialists, seven family specialists, four with hosts in 2–3 families and nine generalists (four or more families or at least two phyla). While the genus and family specialists appeared more likely to show positive test outcomes, the effect of host specialization was likewise not significant (Fisher's exact test: *p* = 0.65), even if genus and family specialists were pooled and compared to more generalist species (Fisher's exact test: *p* = 0.20).

Analysis of related generalist-specialist paired species (see electronic supplementary material) likewise did not show any significant difference, with nine specialist winners, six tied and three generalist winners (binomial test, *p* = 0.24).

### (d) Outcome consistency for species tested more than once

Nine insect species with native and 11 species with non-native hosts were subject to two or more tests of oviposition and survival rates across hosts of varying conditions (table 3). Test outcomes were often inconsistent, especially across different test categories (age, stress, damage and disease). Note that even tests within the same category are not replication studies, as the plant species and type of damage, disease or stressor could vary.

## 4. Discussion

In general, it appears the ‘mother knows best’ prediction can explain insect allocation of eggs across conspecific plants in varying conditions. Some 77% of insects tested with native hosts and 64% of those on non-native hosts allocated eggs to the best or a subset of equal-best hosts for their offspring. This is similar to the results previously obtained for tests across plant species where 83% of insect species showed adaptive oviposition behaviour with their native hosts and 57% with non-native host plant species [5]. It is clear, however, that the ability of ovipositing females to lay eggs on the right host has been tested more extensively for plant species (178 studies on 161 insect species) [5] rather than qualities of individual plants (71 studies on 62 insect species) (see Results). Consequently, we cannot confidently say whether there is a real difference between the strength of intraspecific preference–performance relationships for native and non-native host plants. Likewise, while the smaller gulf between results for native and non-native hosts for intraspecific variation may indicate that some of the cues indicating age, stress, damage or disease are less species specific than the volatile blends used to recognize host species, with the present data we cannot be sure whether a common native range is any less important.

Many authors have predicted that greater specialization would lead to finer tuned adaptations for recognizing suitable larval hosts (e.g. [22–24]), with Bernays [23] introducing the concept of neural constraints on host selection by generalists. These results, however, revealed no significant differences across host range. Indeed, for species tested with native hosts it was the plant family specialists rather than species or genus specialists that were most likely to allocate eggs to the best plant (table 2). However, very few generalists have been tested with their native host species. In Jones *et al*. [5] I defined generalists as feeding across four or more plant families, as used by Bernays & Graham [25]. Among the insects tested with native hosts in this review, however, only two insect species (*Polygonia c-album, Vanessa cardui*) met this definition, with a third, *Samea multiplicalis*, included in the generalist category because its three recorded host families spanned two different plant phyla. These three species showed variable results in different studies (table 3). A total of just five out of 43 species fed on more than one host family.

The situation was very different when it came to tests of exotic host plants. More than half of insect species tested with non-native or crop hosts fed on more than one host family, and nine out of 22 fed on more than three families (table 2). Impressively, eight out of nine (89%) of genus or family specialists allocated eggs to the best or equal-best plant for offspring survival despite the test plants being an exotic species (table 2). This compares to just seven out of 13 (54%) insects with more than one host family (table 2). While the relatively small number of studies meant this difference was not significant, if the effect is real it would suggest that for distinguishing amongst good and poor quality individual plants, exotic hosts are mainly a problem for generalists—for whom a non-native host may be an entirely different family to the plants they are primarily adapted to. While at present there is no strong evidence that generalist species are less likely to allocate their eggs adaptively according to host quality, more data, particularly for generalists on plants from their native range, will be needed to confirm or rule out this hypothesis.

Over the years since the preference–performance hypothesis was first proposed by Wiklund [2], a few contending hypotheses have been proposed, with natural enemies and ‘optimal bad motherhood’ in particular gaining popularity [26,27]. The enemy-free space hypothesis is that insects may evolve to lay eggs on plants that reduce the chance of attack from predators or parasitoids, potentially as a trade-off against nutritional suitability of the plant [26,28]. The most convincing examples of where insects have adapted (or exapted) host plant use for defence against natural enemies are insects that sequester defensive chemicals, rendering them distasteful or poisonous to natural enemies [29,30]. However, both here and in [5], chemical defence sequestration has only been necessary for explaining a case of *few eggs on high survival host,* suggesting the hosts females accept are at least equally good if not better in nutritional value compared to those without the defensive compounds.

In most studies, however, enemy-free space simply means that the most attractive plant or plant stage hosts fewer predators and parasitoids. Two studies in this review found evidence for this (both involving age of the plant) with a native host, whereas a third (involving plant shading) with a non-native host found the attractive host had more predators (but was nutritionally better) (electronic supplementary material). Similarly, in [5], only four studies found enemy-free space, two of them involving non-native hosts, with a fifth finding no trade-off between enemy-free space and laboratory survival. Whether this handful of examples are genuine cases of natural enemies driving evolution of host recognition mechanisms rather than the reduced predation on the attractive host being mere coincidence remains to be seen, but in any case seems an exception rather than a rule. Likewise, only one possible example of ‘optimal bad motherhood’ was found in this review, and not with a native host plant. Like the single example in [5], it involved adult deterrence from nutritious leaves with trichomes rather than females ovipositing on plants that were better adult than larval food sources.

Among the species that had been tested multiple times, it is notable that the same insect species tested for different types of plant variation had the same result no more often than by chance (table 3). Many insect species were capable of distinguishing stressed or damaged plants from healthy ones but not young from old, or vice-versa, or only responded adaptively to certain kinds of herbivore damage or stress, even with the same host species (electronic supplementary material). Plants of different ages can vary greatly in the blend of volatiles they release, whether constitutive or induced [31–33]. Different types of feeding damage and combinations of these have been shown to affect insect olfactory responses to host plants [34]. Volatile profiles in response to herbivore damage can also vary greatly by time of day or night [35].

Likewise, plants infected by bacteria or viruses will also have a unique volatile profile. Many plants release chemicals with antimicrobial properties in response to infection that either function as a volatile to disrupt cellular function of a pathogen, or are defensive compounds that function within the plant tissues that leak out through the cell walls and volatilize, while the pathogens themselves may induce volatiles that attract insect vectors [36].

As well as chemical cues, visual and tactile signals may also provide signs of plant disease, stress or age. For example, the yellow colour of infected pea plants has been shown to attract an aphid vector [37]. Plant or leaf age may be indicated by both visual and tactile cues, such as leaf trichome density that appears to influence oviposition in a leaf miner [38].

Given the complexity of these cues, all of which may indicate differences in the plant's suitability for larval development, and the fact that in the field plants may be attacked by multiple herbivores and endure multiple stressors making behavioural adaptations harder to evolve, it seems unsurprising that ovipositing females are unable to respond perfectly to every situation (see Bernays [23]) and impressive that they can, mostly, detect which plant is in the best condition for offspring development.

## Data Availability

I include all data with the manuscript as electronic supplementary material [39].
